# A functional context for heterogeneity of the circadian clock in cells

**DOI:** 10.1371/journal.pbio.3000927

**Published:** 2020-10-14

**Authors:** Martha Merrow, Mary Harrington

**Affiliations:** 1 Institute of Medical Psychology, Medical Faculty, LMU Munich, Munich, Germany; 2 Neuroscience Program, Smith College, Northampton, Massachusetts, United States of America

## Abstract

Characterization of circadian systems at the organism level—a top-down approach—has led to definition of unifying properties, a hallmark of the science of chronobiology. The next challenge is to use a bottom-up approach to show how the molecular workings of the cellular circadian clock work as building blocks of those properties. We review new studies, including a recently published *PLOS Biology* paper by Nikhil and colleagues, that show how programmed but also stochastic generation of variation in cellular circadian period explain important adaptive features of entrained circadian phase.

## Abstract 2

A recent PLOS Biology paper shows that clonal cell populations are themselves a collection heterogeneous cellular circadian clocks; this Primer explores the implications, proposing that the phase of entrainment of biological clocks (to time of day or to season) is granular, built from the contributions of individual cells.

Circadian clocks provide a temporal structure to most biological processes. These internal circa 24-h clocks entrain to predictable signals in the environment (termed “zeitgebers”) that cue time of day or season (Box 1). The regulation of phase of entrainment contributes the adaptive advantage of the circadian clock [1,2]. It’s the raison d'être of the clock. How does a cellular clock accomplish the entrainment that facilitates adaptive value?

**Box 1. Key characteristics of a circadian clock**

1. Entrainment. All circadian clocks synchronize to zeitgebers. Zeitgebers communicate the 24-h external environment. Entrainment is assessed by entrained phase: the relationship between endogenous and exogenous oscillations. For instance, the onset of melatonin secretion varies between individuals and reflects entrained phase. Because circadian clocks are complex and controlled by many genes, entrained phase in outbred populations shows a distribution. This distribution (chronotype) is influenced by genes, light environment, and development.
2. Free-running period. A remarkable property of most circadian clocks is the ability to continue oscillating even in the absence of a zeitgeber cycle. Internal phase relationships change drastically on the shift from zeitgeber cycles to constant conditions, demonstrating the important role of entrainment on the adaptive function of the circadian clock. The period of the circadian clock in the absence of zeitgebers is approximately but not exactly 24 h.

## Organization of circadian systems

**Fig 1:**
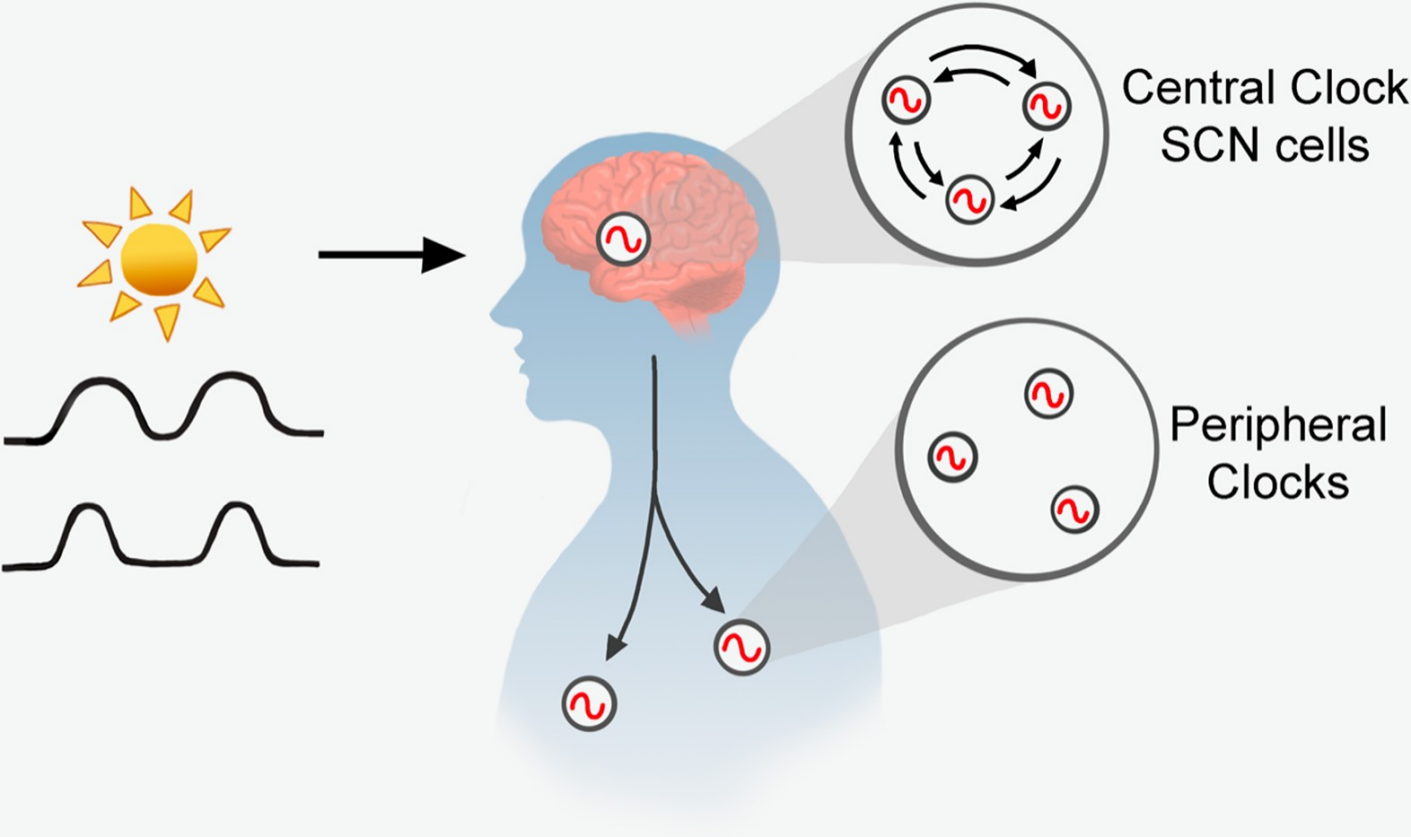
Overview of mammalian circadian organization. See Box 2 for more information. SCN, suprachiasmatic nucleus

Circadian systems (Fig 1) are sometimes compared to an orchestra, with many players that all have to listen to a conductor. For our purposes here, the players are the unique and varied cellular oscillators. The conductor for a cell is its zeitgeber, reliably communicating the pace. Just as players in an orchestra respond according to a specified set of rules, so it is with entrainment of the parts of a circadian system.

The phase to which a circadian clock entrains is related to another property of the daily clock: the period of the free-running rhythm that can be observed in constant environmental conditions (see Box 1). A longer free-running rhythm usually leads to a later entrained phase relative to a shorter one. This has been shown on various levels, demonstrating the pervasiveness of the principles of circadian biology: not only do whole organisms show these properties, but so do the organs and cells within them [3,4].

Phase of entrainment depends on more than just period [5,6]. Another important factor is the strength of the zeitgeber cycle. “Strength” might be determined by the intensity of light or the number of hours per day of sunlight or instead by the sensitivity of the response system to the light. Amplitude of the endogenous oscillation figures in as well.

## Central versus peripheral cellular circadian clocks

All cells are not equal. They each have a context that can mitigate their entrainment to zeitgebers. In mammals, retinal projections signal the light environment to the brain hypothalamic suprachiasmatic nucleus (SCN) (Fig 1). The neuronal oscillators in the SCN show evidence of tight coupling, whereby groups of cells exchange information about circadian phase to generate stable phase relationships [7]. Environmental changes, such as seasonal changes in day length, are reflected in altered phase relationships between the SCN neuronal oscillators [8]. Disruption of neuronal coupling in SCN slices suggests that this tight coupling leads to a narrow range of entrainment [3,9] (see Box 2). This is reflected at the level of behavior. The range of entrainment of mice is circa 21 to 28 h, and that of humans is 22 to 26.5 h [10].

**Box 2. Circadian organization**

Signals from the external environment that reliably represent the external day to the circadian clock (“**zeitgebers**”) cue the body to time of day and changing seasons, here (Fig 1) shown by changes in daylength (“**photoperiod**”). Zeitgebers control the phases of the body rhythms relative to the external day (“**entrainment**”). In the absence of zeitgebers, cells show rhythms of about a day (“**circadian**”) but with a range of cycle lengths (“**circadian periods**”). In most of the body, these cellular clocks do not influence each other’s timing and are “**uncoupled**,” but in a brain region called the suprachiasmatic nucleus (**SCN**), cellular clocks form a coupled oscillator network. Differences in cellular clock structure might be reflected in altered entrainment properties. For instance, SCN clocks entrain to a narrower range of cycle lengths, compared to peripheral cellular clocks. (Peripheral clocks have a wider “**range of entrainment**”).

In peripheral cellular clocks, zeitgebers may come from within or from outside (Fig 1). However, in general, it is the light/dark entrained SCN that drives daily rhythms in physiology and behavior that, in turn, create cellular zeitgebers (e.g., body temperature, food intake, and hormonal outputs). These signals are used by the multiple body clocks to entrain. If critical environmental factors change (as, for example, food becoming scarce or only available at a specific time), body clocks can disengage from the SCN signal and shift their phase according to the new zeitgeber (e.g., food availability) time. Isolated peripheral organs show damping circadian oscillations in vitro. However, cells within these organs can show long-lasting individual cellular oscillations; the key difference appears to be that cells in peripheral organs show little or no cellular coupling [11] so that they drift out of phase with one another.

## Heterogeneity of cellular circadian period

The observation that rhythmicity damps due to oscillatory signals drifting apart implies variability in the free-running period of cells, an observation that holds true for both SCN and peripheral cells [12–15]. Individual cells show a distribution of free-running periods and entrained phases when monitored using reporters of gene expression. A paper published in 2019 in the BioRxiv [16] and in a recent issue of *PLOS Biology* [4] addresses these issues. In addition, papers by Li and colleagues [17,18] report similar findings using different cell lines (immortalized mouse ear fibroblasts versus U-2 OS human osteosarcoma cells versus 3T3 cells), thus suggesting that this is a general property of cellular clocks. Clonal lines could be subcloned to yield short or long period lines—from 22 to 28 h [4,16]. The circadian period of these subclones showed a degree of stability that was—to a modest extent—heritable. The lengthened or shortened periods were not due to spontaneous mutations but rather likely due to modulation of clock gene expression via epigenetic modifications. The case for methylation is particularly strong, and in many cases, these marks can be passed from mother to daughter cell [18]. Indeed, methylation has been indicated in regulating circadian period [19].

These studies show that the occurrence of variability in free-running period is not stochastic in the sense that a cell might be short one day and long the next but rather that (through as-yet-unknown mechanisms) stochastically driven epigenetic modifications predestine a cell to a certain free-running period until those marks are removed. That the circadian clock tolerates stochastic noise [20] should have been a clue that the generation of noise within the system is functional.

## A clock for all seasons?

Why would biological clocks build in mechanisms to change entrainment characteristics from the bottom up? Let us count the ways! The central circadian clock must interpret predictable changes in the light environment. For some mammals, this is translated to an all-or-none reproductive status. Seasonal adjustments of circadian phase are associated with “period aftereffects”: the free-running period following exposure to a long photoperiod differs from that observed following a short photoperiod [21]. Furthermore, changes in methylation of brain cells have been associated with seasonal responses [22]. Period aftereffects in mice held in light/dark cycles of different length (T-cycles) are associated with changes in SCN methylation status [19], leading to changed network properties, reinforcing the idea that the mechanism characterized in subcloned cell lines may be related to the adaptive nature of entrainment. Concerning humans, seasonal changes in, e.g., depression, are well documented, and it is tempting to speculate that the underlying mechanism in the SCN to cope with systematic changes in the light environment may be broken. In exposure to artificial, low-light environments, we may be effectively compromising our “endogenous Markhov chain”—the circadian clock as it predicts the upcoming light environment based on the previous one.

Entrained phase at the behavioral level changes systematically over a lifetime, with adolescents showing a marked delay in the timing of sleep [23]. This suggests an endogenous shift in the interpretation of light as a zeitgeber, but it could just as well be implemented by systematic changes in entrainment characteristics of pacemaker cells or feedbacks on them. We recently suggested that the late chronotype in adolescence may be mediated by Insulin-like Growth Factor–1 [24], which is expressed in puberty at high levels and which induces both advancing and delaying phase shifts in clock gene promoter activity in entrained hypothalamic cells in tissue culture. Interestingly, methylation has also been invoked as a mechanism of mediating IGF-1 expression [25].

Independent of the developmentally dictated change in chronotype, the timing of human behavior is known to have a heritable component. Cracking the genetics of chronotype has been complicated. This may partially be due to the messiness of human behavior, coupled now, as we see, with the changeable clock properties of an individual’s clock system components. Surprisingly, with highly subjective and limited questions, GWAS interrogating hundreds of thousands of genomes yield hundreds of candidate genes as regulators of chronotype. In addition to “the usual suspects” (known clock genes), the list of chronotype-associated genes includes several that regulate methylation, such as TET1, MGMT and METTL15 [26].

## From whence comes precision?

**Fig 2:**
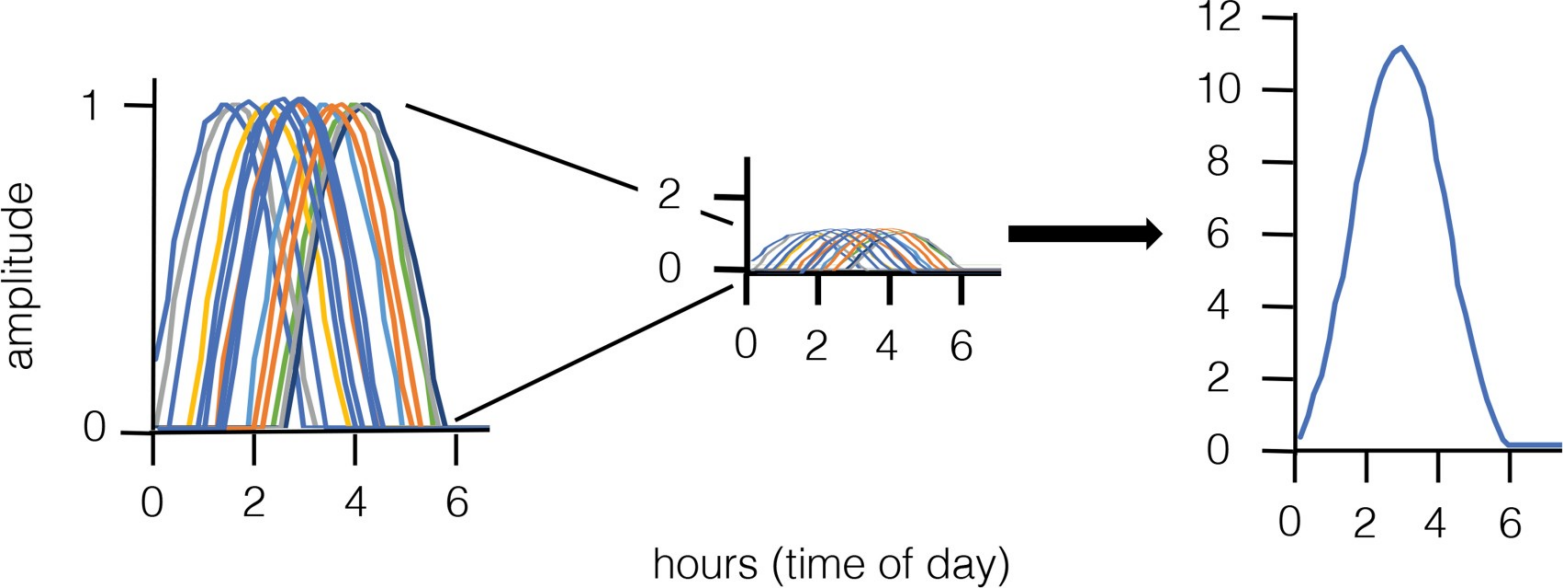
A concept of entrained phase from the level of populations of cells. The curves shown in this figure represent entrained phase to times within the first 6 h of a day (horizontal axis). Apparently, homogeneous cells exhibit a range of free-running periods and therefore a range of entrained phases (left panel, each putative cell line scaled to an amplitude of 1). The center panel depicts the same collection of curves but with the vertical axis scaled proportionally to the panel on the right, which shows a summation of the individual profiles shown to the left. This simply derived additive curve resembles, e.g., bioluminescence profiles showing clock regulated gene expression, such as shown in Nikhil and colleagues [4]. Actual expression curves should vary according to what is quantified—they could be broader or narrower depending on what is measured and on the distribution of component entrained phases.

It is exciting and surprising that these studies on subcloned cells and their interclonal difference suggest a mechanism for determination of chronotype and seasonal responses. This will have implications even into chronopharmacology. But what can we learn about entrainment, which is often called precise, from the collections of periodicities in isolated cells? As stated above, period is an indication of entrained phase, as confirmed by Nikhil and colleagues [1], who showed that the subclones with the longer free-running period entrained up to 5.8 h later than those with the shorter free-running period. A collection of uncoupled cells with various free-running periods will entrain to a range of phases (Fig 2). Entrained phase of the cell culture (and by extension of the organ or organism) is thus a composite of all cells in the population, yielding a broad daily peak. We note that Carr and Whitmore showed this phenomenon in individual zebrafish cells, whereby cells with different periods and amplitudes entrained in light/dark cycles with different relative phase angles [27]. Furthermore, this has already been proposed as a mechanism to facilitate SCN-mediated seasonal behavior [8]. The expression of clock genes, and thus also of those downstream, clock-regulated genes, will be broader rather than narrower over the day under entraining conditions. This is an attractive concept, one which allows that clock-regulated processes are less like a light controlled by an on/off switch and more like one controlled by a dimmer.

What, then, does precision in entrainment mean at a functional level? It is essentially the average of many cellular clocks that must show consistency in phase from day to day. Given that the collection of free-running periods shows a near-normal distribution and that from day to day the period is stable, the collective entrained phase—even though it is represented as a broad peak—will be stable from day to day unless there is an event that induces advances or delays in entrained phase, pushing the collection of cellular periods to be shorter or longer, respectively. The cells with extreme periods will have little impact on the peak phase but rather on the phase of onset or offset. The studies in discussion here noted that the clones with longer free-running periods showed more variance and were less precise from cycle to cycle. These would be predicted to show less-precise entrainment, and this could easily be tested. One would expect that fitting a curve (sine or cosine function) to data of all manner of daily rhythms would show a higher correlation coefficient—over days—to the onset of gene expression in comparison to the offset. If true, this again is a mechanism that is built into the system with some functional correlate.

## The phase-period rule

How organisms find their niche in the environment is the most important physiological function of the circadian clock. If there is a formal relationship between entrained phase and free-running period—some call it a rule—then the observation of a partially heritable, recurring distribution of periods in a clonal cell population might mean that the circadian system engages a range of entrained phases at any given time (Fig 2). The biological clock is built from the bottom up with this feature that likely impacts the clock at all levels. It seems like an important clue as to how nature directed the clock to adapt to a changing environment that is still highly predictable—and that is made much less predictable by the “problem” of our behavior. Evolution was able to accommodate nocturnal and diurnal species and even ground-dwelling animals or those that live above the Arctic Circle. Jet lag and shift work? Not so much.

## Abbreviation

SCN: suprachiasmatic nucleus
